# Supplementary material for: Altered DNA Methylation Profiles in SF3B1 Mutated CLL Patients
Source: Int J Mol Sci. 2021 Aug 28;22(17):9337. doi: 10.3390/ijms22179337 (PMC8431484; doi:10.3390/ijms22179337)
Supplement: Supplementary file 1 [file ijms-22-09337-s001.zip › Supplementary_Material.pdf]

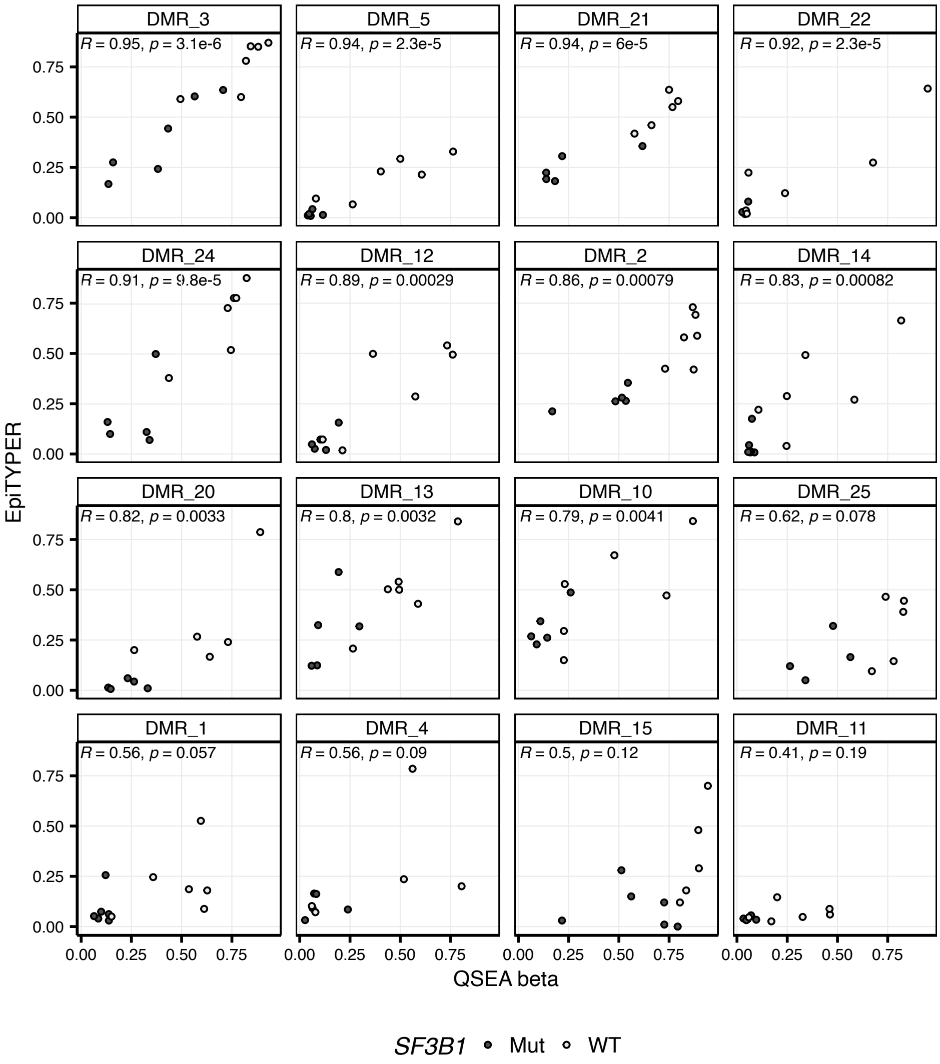

Supplementary Figure S 1. Validation between *SF3B1*^{WT} and *SF3B1*^{mut} CLL patients. Correlation plots between methylation values of CpGs measured with Epityper and QSEA beta values for the 16 DMRs chosen for validation. DMRs were sorted by the correlation p-value.

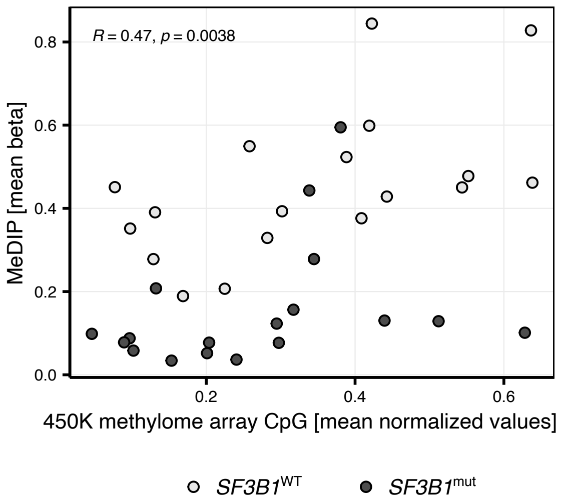

Figure S2. Correlation between mean methylation of 18 DMRs and overlapping CpG from 450K methylome array. Each dot represents a group (CLL *SF3B1*^{WT} or CLL *SF3B1*^{mut}) mean beta methylation value of a DMR detected in this study and corresponding group mean methylation of a CpG located within same region derived from CLLmethylation study and available from BloodCancerMultiOmics2017 R package [1].

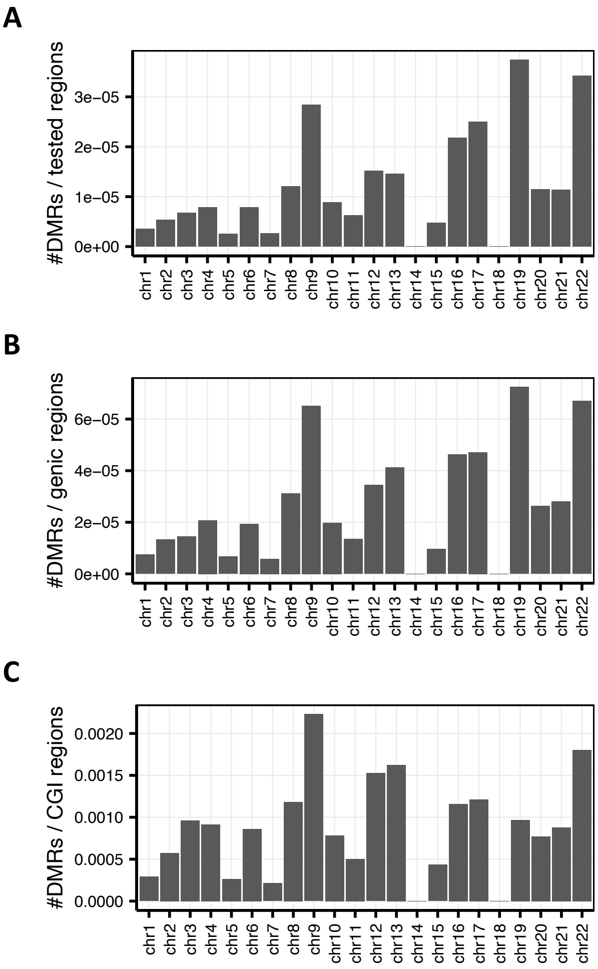

Figure S3. Number of DMRs corrected for A) number of regions tested; B) number of genic regions tested; C) number of regions with a CpG island. The number of differentially methylated regions per chromosome that was identified between *SF3B1*^{WT} and *SF3B1*^{mut} CLL patients' samples divided by the number of A) all regions tested; B) regions that overlapped a gene body; C) regions that overlapped a CpG island at each chromosome.

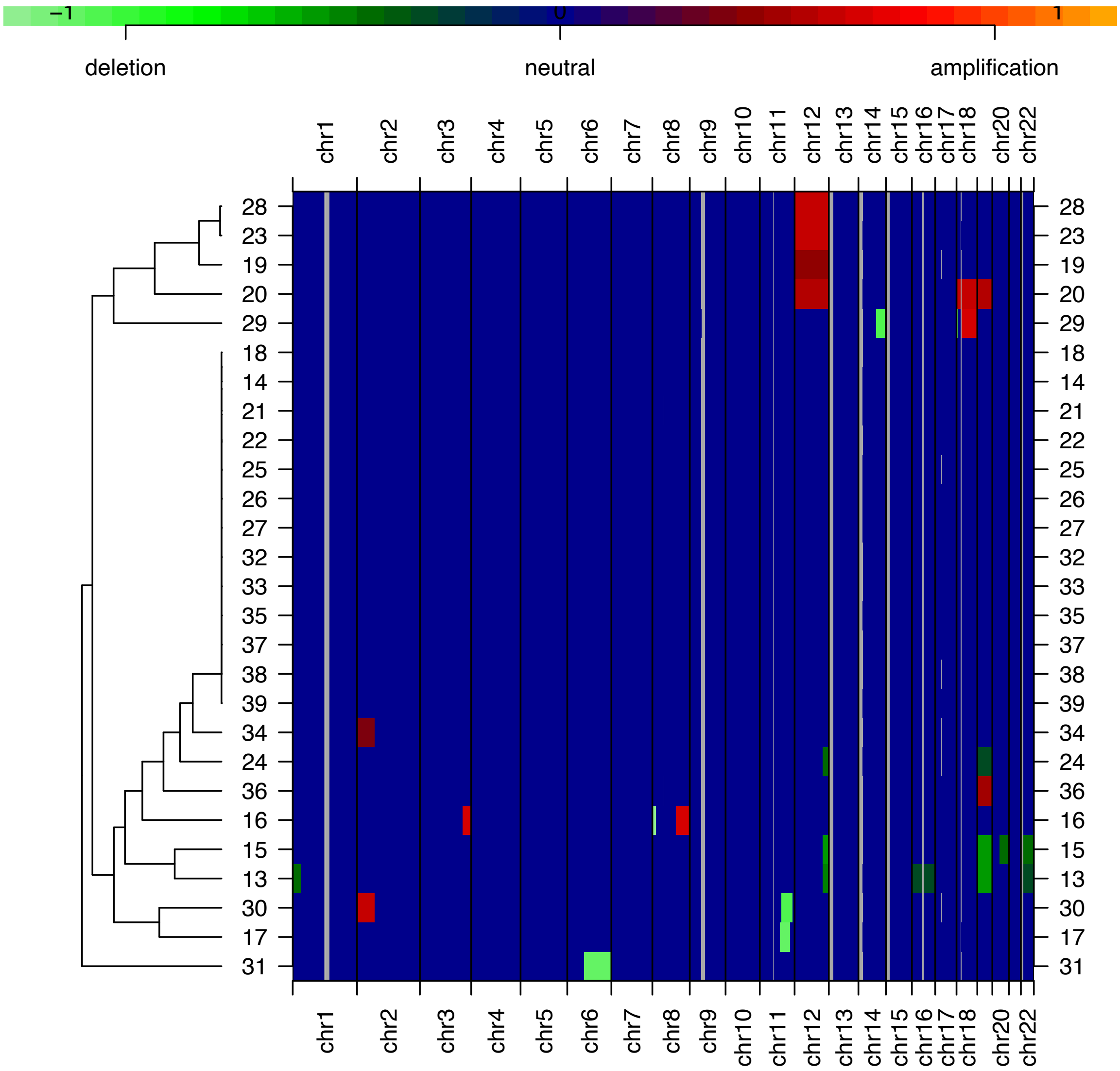

Figure S4. Copy number variation estimated based on MeDIP-seq data. The copy number variation per sample (rows) for each chromosome was calculated with addCNV function of QSEA R package [2] with the following options: window size = 2Mb and fragment size =250bp. The blue color indicates single copy, whereas green indicates a deletion and read an amplification.

A.

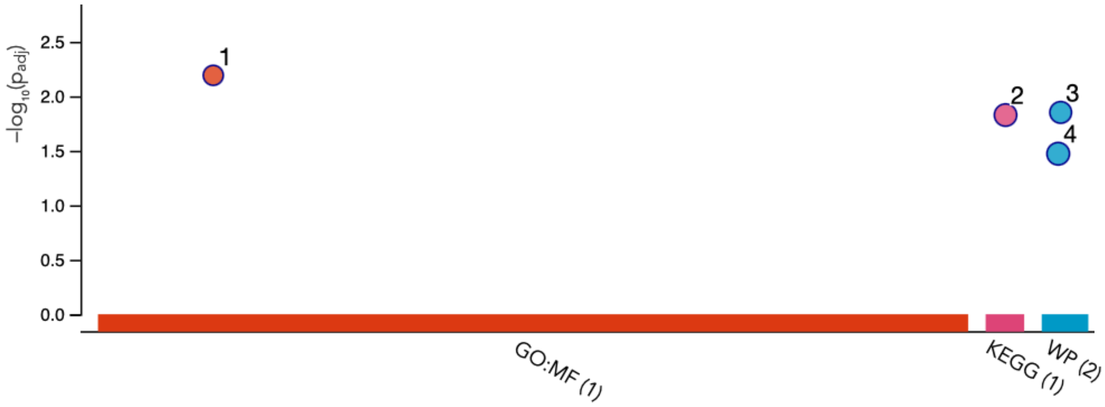

ID	Source	Term ID	Term Name	p_{adj} (query_1)
1	GO:MF	GO:0005112	Notch binding	6.430×10^{-3}
2	KEGG	KEGG:04330	Notch signaling pathway	1.487×10^{-2}
3	WP	WP:WP268	Notch Signaling	1.407×10^{-2}
4	WP	WP:WP61	Notch Signaling Pathway Netpath	3.369×10^{-2}

**version**	e103_eg50_p15_68c0e33	g:Profiler
**date**	13/05/2021, 15:21:35	
**organism**	hsapiens	

B.

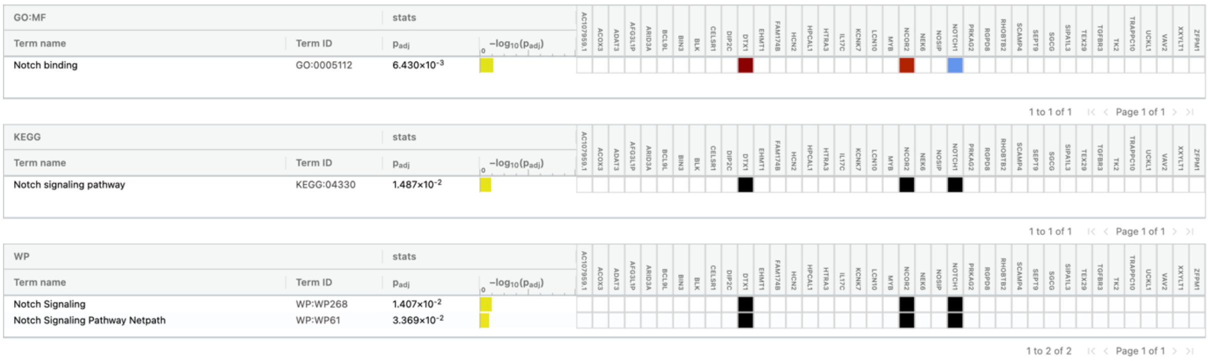

Figure S5. Biological annotation of genes with DMRs. The 39 unique genes that contain a DMR and were annotated were used for enrichment analysis with gProfiler. A) Graphical representation of the enrichment test results with significant terms plotted on the adjusted p-value logarithmic scale. B) Detailed table with the results of the enrichment analysis. All genes taken for the analysis are listed and the boxes are filled if a gene is annotated for the term listed in the first column.

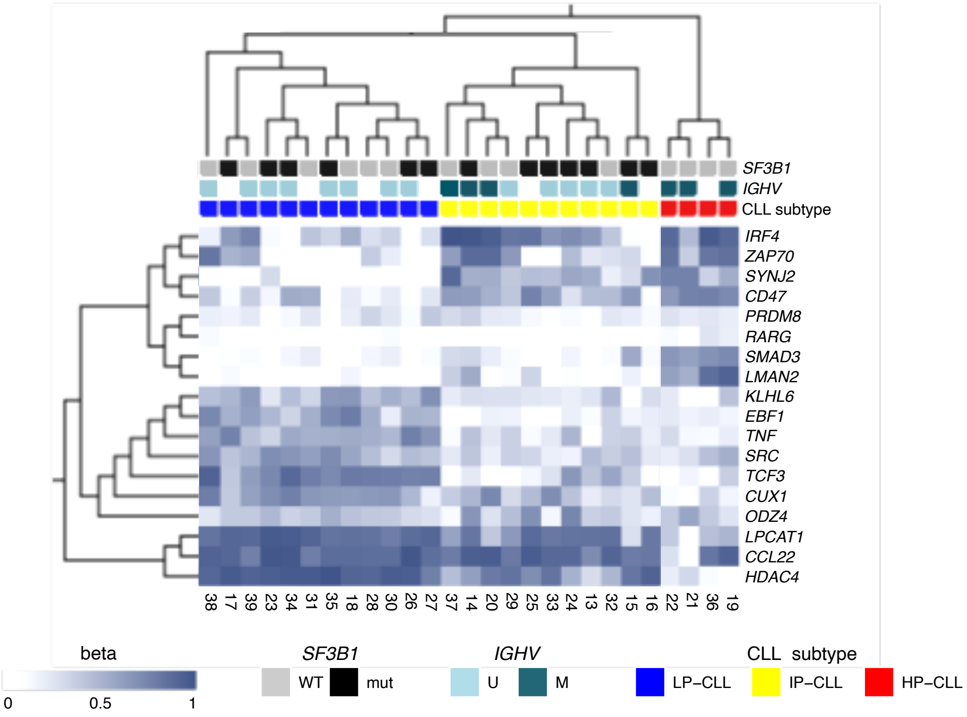

Figure S6. *SF3B1* mutation affects B cells in at least two out of three CLL subtypes. Heat map showing the methylation beta values for the eighteen most variable regions that were used to differentiate CLL subtypes as defined in Oakes et al. [3]. The samples were categorized based on the clusters shown in Figure 4A.

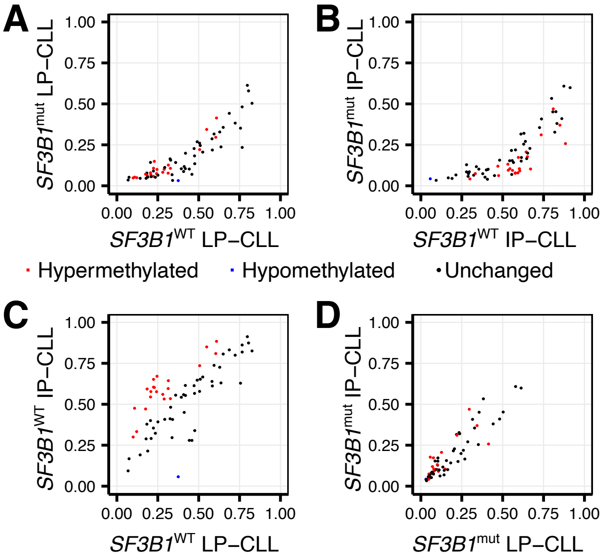

Figure S7. DMRs during physiological B cell maturation. For the 67 differentially methylated regions (DMRs) detected between all *SF3B1*mut and *SF3B1*WT samples we plotted their mean methylation levels (beta normalized) for LP-CLL and IP-CLL samples separately. Every dot represents a DMR. The DMRs are colored by physiological changes during LP- to IP-CLL maturation in *SF3B1*WT - with blue color indicating hypomethylated and red hypermethylated regions (≥ 20% methylation level difference). (A) Comparison between LP-CLL- *SF3B1*mut and LP-CLL- *SF3B1*WT; (B) Comparison between IP-CLL- *SF3B1*mut and IP-CLL- *SF3B1*WT; (C) Comparison between IP-CLL- *SF3B1*WT and LP-CLL- *SF3B1*WT; D) Comparison between IP-CLL- *SF3B1*mut and LP-CLL- *SF3B1*mut.

Table S1. Information about the CLL patients used within the study.

MeDIP ID	MeDIP batch	Binet* disease stage	White blood cells [1/ml]	*SF3B1*	*IGHV*	Sex	Age at sampling [years]	*SF3B1* mutation**	*TP53* mutation**	*ATM* mutation**	*XPO1* mutation**	*NOTCH1* mutation**
18	1	C	49.29	WT	U	male	50					
19	1	B/C	98.89	WT	M	male	64					p.F1606L (5%)
20	1	n.a.	24.3	WT	M	male	48					
21	1	n.a.	24.39	WT	M	female	57					
22	1	A/B	31.62	WT	M	male	60					
28	2	A/B	49.26	WT	N/A	male	74					
29	2	A/B	45.19	WT	U	male	68					
30	2	A/B	52.46	WT	U	male	67					
31	2	A/B	58.5	WT	N/A	male	47					
32	2	n.a.	78.42	WT	U	male	70					
36	3	n.a.	27.64	WT	N/A	male	65					
37	3	A/B	48.71	WT	M	male	57					
38	3	A/B	48.87	WT	U	female	76					
39	3	n.a.	17.85	WT	U	male	64					
13	1	A/B	124.02	mut	U	male	52	H662Q (45%)				
14	1	A/B	149.42	mut	M	male	64	K700E (44%)				
15	1	A/B	34.91	mut	M	male	49	G742D (40%)				
16	1	A/B	483.77	mut	N/A	male	60	E622D (49%)				
17	1	A/B	45.81	mut	N/A	male	57	K666M (48%)			p.E571K (49%)	
23	2	A/B	59.92	mut	U	male	75	Q699E (51%)				p.P2514fs (12%)
24	2	C	140.33	mut***	U	male	66	A284T (49%)***				
25	2	C	33.3	mut	N/A	female	74	K700E (48%)				
26	2	A/B	26.69	mut	U	male	69	N626Y (48%)				
27	2	C	51.15	mut	N/A	male	49	D894G (54%); I704N (19%)				
33	3	C	110.26	mut	U	male	72	Y623C (42%)				
34	3	A/B	73.62	mut	U	male	59	I704F (48%)	p.L43**** (6%)			
35	3	A/B	32.76	mut	U	male	64	K666E (51%)		p.C1736Y (66%)		

* Binet's disease stage as defined in Binet *et al*. [4]
** Mutation identified in the patient by Vollbrecht *et al.* 2015 [5]
*** The mutation was identified in the N-terminal, outside the highly conserved HEAT repeats 5–8, see Fig. 2 legend in Vollbrecht *et al.* 2015 [5]
**** Deletion on the second allele (see Vollbrecht *et al.* 2015 [5])

Table S3. Primers designed for the DMR validation with EpiTYPER MassARRAY

Name	Primer plus tag (small letters)	Anealing temperature [°C]	gene	length of Epityper target	genomic coordinate of Epityper assay	genomic coordinate of DMR from MeDIP-seq
DMR_01_10F	aggaagagagGAGTAGTTGGGATTATAGGTATTTGTT	56		257	chr12:1602716-1602972	chr12:1602751-1603000
DMR_01_T7R	cagtaatacgactcactatagggagaaggctCATCAAAAAACCCTCAAATTTATTC					
DMR_02_10F	aggaagagagGAGATTTATTTGTTTTTTGTTTTAAGGG	60	UCKL1	264	chr20:63950750-63951013	chr20:63950751-63951000
DMR_02_T7R	cagtaatacgactcactatagggagaaggctACTACCCACAAAAATAATCCTACCC					
DMR_03_10F	aggaagagagAAGATTTTTTTAGAATTTTGGGTTA	56	ACOX3	290	chr4:8384511-8384800	chr4:8384501-8384750
DMR_03_T7R	cagtaatacgactcactatagggagaaggctTCCAAACCTTACTTTCCAAAACTTA					
DMR_04_10F	aggaagagagGTTTTTTATTTAGGGTGGGGTATTT	56	BCL9L	480	chr11:118910355-118910834	chr11:118910501-118910750
DMR_04_T7R	cagtaatacgactcactatagggagaaggctAATCTCTAAACCTCTACTAACAACACC					
DMR_05_10F	aggaagagagGTGGGGTTGTTGTAGGGTATTTT	56	ZFPM1	374	chr16:88478366-88478739	chr16:88478501-88478750
DMR_05_T7R	cagtaatacgactcactatagggagaaggctAATTAAAACCTCCTCATCCCTCTAA					
DMR_10_10F	aggaagagagTTGTTAGTTTGTGTTTAGGTAGGGG	56	SEPTIN9	422	chr17:77435444-77435865	chr17:77435501-77435750
DMR_10_T7R	cagtaatacgactcactatagggagaaggctAACTCAAAAACAAAAACCTCCAAAT					
DMR_11_10F	aggaagagagGATTTTTGTAATTGTTGTTTGGTGT	60	HTRA3	290	chr4:8270104-8270393	chr4:8270001-8270250
DMR_11_T7R	cagtaatacgactcactatagggagaaggctAAAAAAAACTTCACTTCCTCCCTAA					
DMR_12_10F	aggaagagagTGTTTATTTAAGTTGGTGTAGGGGA	56	SGCG	391	chr13:23324392-23324782	chr13:23324501-23324750
DMR_12_T7R	cagtaatacgactcactatagggagaaggctTCCAATAAAAAAACACATTACTTTTCC					
DMR_13_10F	aggaagagagAGAGTTGGGGGTTTTATTAGTTTTTT	56	SCAMP4; ADAT3	465	chr19:1909389-1909853	chr19:1909501-1909750
DMR_13_T7R	cagtaatacgactcactatagggagaaggctTCCCTTAACCACATTCCTACAACTA					
DMR_14_10F	aggaagagagAAAGAGTGGAGGTTAGGAGAGTTGT	56	RHOBTB2	344	chr8:23003905-23004248	chr8:23004001-23004250
DMR_14_T7R	cagtaatacgactcactatagggagaaggctCAAAAACCCATTCAAACTAAAAAAA					
DMR_15_10F	aggaagagagTATGTGTTAGTGTGTAAGATTGTGTGTAA	60	CELSR1	218	chr22:46420444-46420661	chr22:46420251-46420500
DMR_15_T7R	cagtaatacgactcactatagggagaaggctACATACTCACCCAAATACACTCACC					
DMR_20_10F	aggaagagagGTTTTGTTGTTTAGGTTGGAGTGTAG	60	TK2	413	chr16:66526917-66527329	chr16:66527001-66527250
DMR_20_T7R	cagtaatacgactcactatagggagaaggctCAAAATAAAAACCTCTACCCCAAAT					
DMR_21_10F	aggaagagagAGTATTTTTGTTGGGTATTATGGTT	56	HPCAL1	433	chr2:10325839-10326271	chr2:10326001-10326250
DMR_21_T7R	cagtaatacgactcactatagggagaaggctAAAACAAAAAAATCTCCCATCTACC					
DMR_22_10F	aggaagagagTTTTTAGGAGGATAGGGTAGGGTTA	56	IL17C	198	chr16:88639754-88639951	chr16:88639751-88640000
DMR_22_T7R	cagtaatacgactcactatagggagaaggctACAAAAACCCCAAATATAAAAAACC					
DMR_24_10F	aggaagagagTTAGTTTTGTGTTTATTGAGGGAGG	56	BLK	474	chr8:11517930-11518403	chr8:11518001-11518250
DMR_24_T7R	cagtaatacgactcactatagggagaaggctTTCAAAATCAAAACCCAATATCTAAC					
DMR_25_10F	aggaagagagGTGTGGGATTTTAGGTTAGGGTTTA	60	BIN3	353	chr8:22627450-22627802	chr8:22627501-22627750
DMR_25_T7R	cagtaatacgactcactatagggagaaggctCTATCTAACCTACCCCCACCTAACC					

Table S4. Epityper methylation values compared to MeDIP-seq beta values. Methylation values are given for individual samples. Shown are the MeDIP-seq beta values and the mean EpiTYPER methylation values for a given amplicon. In addition, the methylation values for the individual CpG units within an amplicon, that passed the quality criteria are shown. Excluded were CpG units with more than 2 CpGs, a bad standard curve, with less than 50% of the samples having methylation values, CpG units with at least two peaks underlying the unmethylated CpG unit and CpG units with identical masses. Cells are colored by the methylation values for individual CpGs.

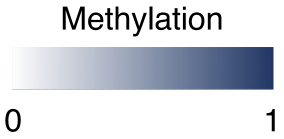

DMR	sample	SF3B1	MeDIP beta	EpiTYPER mean	CpG position in amplicon				
					42	94	132	177	212
DMR_1	19	WT	0.60	0.53	0.63	0.11	0.31	0.79	0.79
DMR_1	21	WT	0.36	0.25	0.31	0.06	0.03	0.74	0.09
DMR_1	28	WT	0.54	0.19	0.04	0.05	0.00	0.42	0.42
DMR_1	30	WT	0.15	0.05	0.07	0.07	0.06	0.04	0.01
DMR_1	31	WT	0.61	0.09	0.06	0.05	0.00	0.16	0.17
DMR_1	36	WT	0.63	0.18	0.42	0.07	0.04	0.28	0.09
DMR_1	14	Mut	0.12	0.26	0.48	0.04	0.05	0.44	0.27
DMR_1	17	Mut	0.10	0.07	0.03	0.06	0.02	0.02	0.24
DMR_1	24	Mut	0.14	0.03	0.02	0.07	0.00	0.00	0.06
DMR_1	25	Mut	0.09	0.04	0.03	0.05	0.02	0.04	0.06
DMR_1	26	Mut	0.14	0.06	0.04	0.05	0.03	0.03	0.16
DMR_1	33	Mut	0.06	0.05	0.06	0.05	0.03	0.06	0.06

DMR	sample	SF3B1	MeDIP beta	EpiTYPER mean	CpG position in amplicon				
					41	99	143	167	225
DMR_2	19	WT	0.87	0.42	0.91	0.36	0.26	0.32	0.25
DMR_2	21	WT	0.73	0.42	0.56	0.60	0.17	0.41	0.38
DMR_2	28	WT	0.87	0.73	0.83	0.64	0.69	0.78	0.71
DMR_2	30	WT	0.82	0.58	0.89	0.56	0.40	0.54	0.51
DMR_2	31	WT	0.89	0.59	0.46	0.64	0.42	0.65	0.77
DMR_2	36	WT	0.88	0.69	0.86	0.68	0.55	0.63	0.74
DMR_2	14	Mut	0.54	0.35	0.77	0.33	0.16	0.14	0.37
DMR_2	17	Mut	0.17	0.21	1.00	0.00	0.00	0.06	0.00
DMR_2	24	Mut	0.48	0.26	0.83	0.38	0.04	0.03	0.03
DMR_2	25	Mut	0.52	NA	NA	NA	NA	NA	NA
DMR_2	26	Mut	0.53	0.26	0.72	0.04	0.04	0.11	0.41
DMR_2	33	Mut	0.52	0.28	0.73	0.26	0.10	0.09	0.22

DMR	sample	SF3B1	MeDIP beta	EpiTYPER mean	CpG position in amplicon			
					74	90	171	235
DMR_3	19	WT	0.93	0.87	NA	0.92	0.78	0.91
DMR_3	21	WT	0.84	0.85	0.73	0.91	0.83	0.94
DMR_3	28	WT	0.82	0.78	0.58	0.79	0.82	0.93
DMR_3	30	WT	0.80	0.60	0.32	0.67	0.60	0.81
DMR_3	31	WT	0.49	0.59	NA	0.41	0.54	0.82
DMR_3	36	WT	0.88	0.85	0.72	0.89	0.85	0.94
DMR_3	14	Mut	0.71	0.64	0.39	0.67	0.63	0.85
DMR_3	17	Mut	0.16	0.28	0.23	0.25	0.33	0.29
DMR_3	24	Mut	0.56	0.60	NA	0.21	0.73	0.87
DMR_3	25	Mut	0.14	0.17	0.00	0.15	0.22	0.30
DMR_3	26	Mut	0.38	0.24	0.14	0.12	0.31	0.40
DMR_3	33	Mut	0.43	0.44	NA	0.36	0.46	0.51

DMR	sample	SF3B1	MeDIP beta	EpiTYPER mean	CpG position in amplicon											
					38	78	125	145:149	211:219	231	282:291	301:305	370	416	455	462
DMR_4	19	WT	0.73	NA	NA	NA	NA	NA	NA	NA	NA	NA	NA	NA	NA	NA
DMR_4	21	WT	0.56	0.77	0.57	0.85	1.00	0.92	0.81	0.76	0.70	0.66	0.93	0.82	0.79	0.43
DMR_4	28	WT	0.08	0.05	0.03	0.02	0.07	0.02	0.00	0.18	0.07	0.02	0.06	0.08	0.04	0.05
DMR_4	30	WT	0.52	0.12	0.00	0.06	0.00	0.10	0.01	0.12	0.05	0.01	0.44	0.55	0.03	0.07
DMR_4	31	WT	0.06	0.07	0.03	0.03	0.00	0.04	0.00	0.09	0.07	0.01	0.04	0.47	0.00	0.03
DMR_4	36	WT	0.81	0.19	0.16	0.05	0.07	0.37	0.02	0.20	0.14	0.12	0.35	0.56	0.00	0.29
DMR_4	14	Mut	0.24	0.09	0.04	0.04	0.29	0.28	0.02	0.12	0.10	0.05	0.06	0.02	0.00	0.02
DMR_4	17	Mut	0.07	0.12	0.34	0.05	0.00	0.02	0.02	0.18	0.07	0.08	0.36	0.31	0.00	0.03
DMR_4	24	Mut	0.06	0.07	0.02	0.06	0.00	0.01	0.02	0.26	0.07	0.03	0.04	0.26	0.00	0.03
DMR_4	25	Mut	0.06	NA	NA	NA	NA	NA	NA	NA	NA	NA	NA	NA	NA	NA
DMR_4	26	Mut	0.08	0.15	0.12	0.18	0.02	0.54	0.00	0.20	0.12	0.03	0.13	0.43	0.00	0.05
DMR_4	33	Mut	0.03	0.02	0.07	0.04	0.00	0.03	0.01	0.00	0.03	0.00	0.00	0.04	0.00	0.02

DMR	sample	SF3B1	MeDIP beta	EpiTYPER mean	CpG position in amplicon							
					59	90:96	140	159	212	251	287	358
DMR_5	19	WT	0.76	0.33	0.32	0.37	0.34	0.42	0.40	0.05	0.32	0.41
DMR_5	21	WT	0.26	0.07	0.01	0.02	0.03	0.03	0.04	0.12	0.11	0.17
DMR_5	28	WT	0.50	0.29	NA	0.14	0.26	0.25	0.26	0.44	0.33	0.37
DMR_5	30	WT	0.40	0.23	0.25	0.04	0.24	0.24	0.24	0.32	0.24	0.27
DMR_5	31	WT	0.61	0.21	NA	0.07	0.26	0.18	0.26	0.25	0.18	0.30
DMR_5	36	WT	0.08	0.10	0.00	0.10	0.06	0.06	0.04	0.24	0.12	0.14
DMR_5	14	Mut	0.04	0.01	0.02	0.01	0.03	0.00	0.03	0.00	0.00	0.00
DMR_5	17	Mut	0.05	0.01	0.00	0.00	0.03	0.01	0.03	0.00	0.00	0.00
DMR_5	24	Mut	0.05	0.02	0.08	0.00	0.01	0.02	0.04	0.00	0.00	0.00
DMR_5	25	Mut	0.05	NA	NA	NA	NA	NA	NA	NA	NA	NA
DMR_5	26	Mut	0.12	0.01	0.00	0.00	0.02	0.00	0.03	0.00	0.00	0.06
DMR_5	33	Mut	0.06	0.04	0.00	0.01	0.04	0.01	0.04	0.22	0.00	0.02

DMR	sample	SF3B1	MeDIP beta	EpiTYPER mean	CpG position in amplicon					
					81	228	247	347	394:397	404
DMR_10	19	WT	0.87	0.84	0.74	0.83	0.96	0.82	0.81	0.89
DMR_10	21	WT	0.48	0.67	0.06	0.67	0.58	0.89	0.83	1.00
DMR_10	28	WT	0.23	0.53	0.05	0.35	0.23	0.64	0.90	1.00
DMR_10	30	WT	0.23	0.15	0.03	0.01	0.03	0.07	0.45	0.31
DMR_10	31	WT	0.23	0.30	0.03	0.04	0.04	0.25	0.69	0.72
DMR_10	36	WT	0.74	0.47	0.04	0.24	0.23	0.77	0.77	0.78
DMR_10	14	Mut	0.26	0.49	0.02	0.21	0.19	0.73	0.92	0.85
DMR_10	17	Mut	0.14	0.26	0.03	0.00	0.05	0.20	0.65	0.64
DMR_10	24	Mut	0.06	0.27	0.04	0.00	0.03	0.11	0.71	0.72
DMR_10	25	Mut	0.12	NA	NA	NA	NA	NA	NA	NA
DMR_10	26	Mut	0.11	0.34	0.06	0.00	0.02	0.12	0.86	1.00
DMR_10	33	Mut	0.09	0.23	0.04	0.00	0.03	0.02	0.66	0.62

DMR	sample	SF3B1	MeDIP beta	EpiTYPER mean	CpG position in amplicon				
					64	136:139	217	247:255	266:270
DMR_11	19	WT	0.46	0.06	0.05	0.06	0.08	0.02	0.09
DMR_11	21	WT	0.46	0.09	0.08	0.10	0.10	0.03	0.13
DMR_11	28	WT	0.20	0.15	0.05	0.07	0.43	0.06	0.12
DMR_11	30	WT	0.17	0.03	0.03	0.02	0.00	0.03	0.05
DMR_11	31	WT	0.06	0.05	0.03	0.03	0.03	0.03	0.11
DMR_11	36	WT	0.33	0.05	0.04	0.03	0.04	0.03	0.10
DMR_11	14	Mut	0.05	0.04	0.03	0.03	0.05	0.02	0.07
DMR_11	17	Mut	0.07	0.06	0.05	0.03	0.01	0.02	0.17
DMR_11	24	Mut	0.10	0.03	0.03	0.04	0.00	0.02	0.08
DMR_11	25	Mut	0.03	0.04	0.05	0.04	0.01	0.01	0.09
DMR_11	26	Mut	0.05	0.04	0.04	0.03	0.00	0.02	0.09
DMR_11	33	Mut	0.05	0.03	0.03	0.02	0.00	0.02	0.09

DMR	sample	SF3B1	MeDIP beta	EpiTYPER mean	CpG position in amplicon				
					36	115	169:177	195	268
DMR_12	19	WT	0.73	0.54	0.68	0.49	0.59	0.52	0.42
DMR_12	21	WT	0.36	0.50	0.06	0.67	0.78	0.87	0.11
DMR_12	28	WT	0.58	0.29	0.30	0.09	0.37	0.55	0.12
DMR_12	30	WT	0.11	0.07	0.06	0.07	0.11	0.12	0.00
DMR_12	31	WT	0.21	0.02	0.01	0.04	0.04	0.00	0.00
DMR_12	36	WT	0.76	0.49	0.40	0.41	0.57	0.71	0.38
DMR_12	14	Mut	0.19	0.16	0.09	0.15	0.18	0.28	0.08
DMR_12	17	Mut	0.07	0.03	0.04	0.03	0.05	0.01	0.00
DMR_12	24	Mut	0.06	0.05	0.02	0.08	0.06	0.08	0.00
DMR_12	25	Mut	0.06	NA	NA	NA	NA	NA	NA
DMR_12	26	Mut	0.10	0.07	0.02	0.03	0.04	0.00	0.27
DMR_12	33	Mut	0.13	0.02	0.03	0.03	0.04	0.00	0.00

DMR	sample	SF3B1	MeDIP beta	EpiTYPER mean	CpG position in amplicon				
					162	291	308	336	358
DMR_13	19	WT	0.79	0.84	0.92	0.99	0.90	0.45	0.94
DMR_13	21	WT	0.44	0.50	0.60	0.28	0.27	NA	0.86
DMR_13	28	WT	0.26	0.21	0.21	0.27	0.26	NA	0.09
DMR_13	30	WT	0.49	0.54	0.43	0.79	0.83	0.52	0.13
DMR_13	31	WT	0.49	0.50	0.67	0.45	0.38	0.45	0.55
DMR_13	36	WT	0.59	0.43	0.48	0.54	0.47	0.00	0.66
DMR_13	14	Mut	0.30	0.32	0.39	0.26	0.09	0.00	0.85
DMR_13	17	Mut	0.09	0.32	0.31	0.13	0.36	0.00	0.82
DMR_13	24	Mut	0.09	0.12	0.06	0.04	0.13	0.04	0.35
DMR_13	25	Mut	0.08	NA	NA	NA	NA	NA	NA
DMR_13	26	Mut	0.19	0.59	0.11	0.38	0.62	1.00	0.83
DMR_13	33	Mut	0.06	0.12	0.18	0.03	0.00	0.00	0.40

DMR	sample	SF3B1	MeDIP beta	EpiTYPER mean	CpG position in amplicon				
					106:112	160	225	247:249	286
DMR_14	19	WT	0.11	0.22	0.00	0.01	0.25	0.37	0.47
DMR_14	21	WT	0.82	0.66	0.13	0.40	0.89	0.90	1.00
DMR_14	28	WT	0.34	0.49	0.02	0.18	0.55	0.71	1.00
DMR_14	30	WT	0.25	0.04	0.00	0.01	0.03	0.07	0.09
DMR_14	31	WT	0.25	0.29	0.00	0.01	0.40	0.51	0.52
DMR_14	36	WT	0.58	0.27	0.02	0.02	0.23	0.44	0.64
DMR_14	14	Mut	0.06	0.04	0.00	0.01	0.09	0.03	0.09
DMR_14	17	Mut	0.09	0.01	0.00	0.01	0.00	0.02	0.01
DMR_14	24	Mut	0.07	0.01	0.00	0.02	0.03	0.01	0.00
DMR_14	25	Mut	0.07	0.18	0.52	0.00	0.18	0.00	NA
DMR_14	26	Mut	0.07	0.01	0.00	0.02	0.00	0.02	0.00
DMR_14	33	Mut	0.06	0.01	0.00	0.01	0.02	0.02	0.00

DMR	sample	SF3B1	MeDIP beta	EpiTYPER mean	CpG position in amplicon
					172
DMR_15	19	WT	0.94	0.70	0.70
DMR_15	21	WT	0.90	0.48	0.48
DMR_15	28	WT	0.80	0.12	0.12
DMR_15	30	WT	0.83	0.18	0.18
DMR_15	31	WT	0.90	NA	NA
DMR_15	36	WT	0.90	0.29	0.29
DMR_15	14	Mut	0.73	0.12	0.12
DMR_15	17	Mut	0.73	0.01	0.01
DMR_15	24	Mut	0.22	0.03	0.03
DMR_15	25	Mut	0.79	0.00	0.00
DMR_15	26	Mut	0.51	0.28	0.28
DMR_15	33	Mut	0.56	0.15	0.15

DMR	sample	SF3B1	MeDIP beta	EpiTYPER mean	CpG position in amplicon		
					48	65:70	257
DMR_20	19	WT	0.89	0.79	0.80	0.80	0.76
DMR_20	21	WT	0.64	0.17	0.19	0.14	0.17
DMR_20	28	WT	0.73	0.24	0.28	0.25	0.19
DMR_20	30	WT	0.26	0.20	0.21	0.21	0.18
DMR_20	31	WT	0.58	0.27	0.32	0.38	0.10
DMR_20	36	WT	NA	0.38	0.46	0.37	0.31
DMR_20	14	Mut	0.13	0.01	0.00	0.01	0.03
DMR_20	17	Mut	0.33	0.01	0.01	0.01	0.01
DMR_20	24	Mut	0.15	0.01	0.00	0.02	0.00
DMR_20	25	Mut	0.30	NA	NA	NA	NA
DMR_20	26	Mut	0.26	0.04	0.02	0.04	0.07
DMR_20	33	Mut	0.23	0.06	0.10	0.07	0.01

DMR	sample	SF3B1	MeDIP beta	EpiTYPER mean	CpG position in amplicon				
					57	244	257	275	371:375
DMR_21	19	WT	0.75	0.64	0.25	0.84	0.89	0.65	0.55
DMR_21	21	WT	0.79	0.58	0.05	0.84	0.73	0.76	0.52
DMR_21	28	WT	0.77	0.55	0.06	0.53	0.79	0.63	0.74
DMR_21	30	WT	0.58	0.42	0.04	0.52	0.64	0.58	0.31
DMR_21	31	WT	0.66	0.46	0.08	0.62	0.71	0.35	0.54
DMR_21	36	WT	0.74	NA	NA	NA	NA	NA	NA
DMR_21	14	Mut	0.62	0.36	0.03	0.55	0.66	0.35	0.19
DMR_21	17	Mut	0.18	0.18	0.08	0.28	0.35	0.15	0.05
DMR_21	24	Mut	0.14	0.22	0.04	0.16	0.59	0.30	0.03
DMR_21	25	Mut	0.16	NA	NA	NA	NA	NA	NA
DMR_21	26	Mut	0.14	0.19	0.02	0.37	0.28	0.26	0.03
DMR_21	33	Mut	0.22	0.31	0.05	0.44	0.61	0.29	0.14

DMR	sample	SF3B1	MeDIP beta	EpiTYPER mean	CpG position in amplicon				
					54:49	106:109	162	172:176	226:231
DMR_22	19	WT	0.04	0.04	0.03	0.04	0.07	0.02	0.02
DMR_22	21	WT	0.05	0.02	0.03	0.04	0.00	0.02	0.01
DMR_22	28	WT	0.95	0.64	0.76	0.66	0.88	0.62	0.29
DMR_22	30	WT	0.24	0.12	0.38	0.14	0.01	0.06	0.02
DMR_22	31	WT	0.68	0.27	0.58	0.12	0.38	0.17	0.12
DMR_22	36	WT	0.06	0.22	0.23	0.21	0.37	0.25	0.06
DMR_22	14	Mut	0.06	0.08	0.25	0.05	0.04	0.03	0.03
DMR_22	17	Mut	0.04	0.02	0.02	0.03	0.00	0.02	0.02
DMR_22	24	Mut	0.04	0.03	0.04	0.05	0.01	0.02	0.01
DMR_22	25	Mut	0.03	0.03	0.04	0.02	0.04	0.02	0.02
DMR_22	26	Mut	0.03	0.03	0.04	0.02	0.03	0.02	0.02
DMR_22	33	Mut	0.03	0.03	0.03	0.04	0.00	0.04	0.03

DMR	sample	SF3B1	MeDIP beta	EpiTYPER mean	CpG position in amplicon
					404
DMR_24	19	WT	0.82	0.88	0.88
DMR_24	21	WT	0.76	0.78	0.78
DMR_24	28	WT	0.77	0.78	0.78
DMR_24	30	WT	0.73	0.73	0.73
DMR_24	31	WT	0.44	0.38	0.38
DMR_24	36	WT	0.75	0.52	0.52
DMR_24	14	Mut	0.13	0.16	0.16
DMR_24	17	Mut	0.33	0.11	0.11
DMR_24	24	Mut	0.37	0.50	0.50
DMR_24	25	Mut	0.16	NA	NA
DMR_24	26	Mut	0.14	0.10	0.10
DMR_24	33	Mut	0.34	0.07	0.07

DMR	sample	SF3B1	MeDIP beta	EpiTYPER mean	CpG position in amplicon	
					75	336
DMR_25	19	WT	0.83	0.39	0.78	0.00
DMR_25	21	WT	0.83	0.45	0.82	0.07
DMR_25	28	WT	0.78	0.15	0.29	0.00
DMR_25	30	WT	0.67	0.10	0.15	0.04
DMR_25	31	WT	0.74	0.47	0.93	0.00
DMR_25	36	WT	NA	0.31	0.48	0.13
DMR_25	14	Mut	0.48	0.32	0.55	0.09
DMR_25	17	Mut	0.34	0.05	0.10	0.00
DMR_25	24	Mut	0.26	0.12	0.22	0.02
DMR_25	25	Mut	0.17	NA	NA	NA
DMR_25	26	Mut	0.40	NA	NA	NA
DMR_25	33	Mut	0.56	0.17	0.19	0.14

Table S5. Validation of MeDIP-seq results by EpiTYPER MASSarray. Given are the mean EpiTYPER methylation values and the mean beta-values calculated from MeDIP-seq by QSEA. The six samples analysed by EpiTYPER are a subset of the samples used for MeDIP-seq.

DMR	EpiTYPER means		MeDIP beta means	
	SF3B1 WT (6 samples)	SF3B1 mut (6 samples)	SF3B1 WT (14 samples)	SF3B1 mut (13 samples)
DMR_1	0.21	0.09	0.48	0.11
DMR_2	0.57	0.27	0.84	0.46
DMR_3	0.76	0.39	0.79	0.40
DMR_4	0.28	0.11	0.40	0.10
DMR_5	0.20	0.02	0.44	0.06
DMR_10	0.49	0.32	0.46	0.13
DMR_11	0.07	0.04	0.28	0.06
DMR_12	0.32	0.06	0.46	0.11
DMR_13	0.50	0.30	0.51	0.15
DMR_14	0.33	0.04	0.39	0.07
DMR_15	0.35	0.10	0.88	0.59
DMR_20	0.33	0.03	0.62	0.22
DMR_21	0.53	0.25	0.71	0.26
DMR_22	0.22	0.03	0.34	0.04
DMR_24	0.68	0.19	0.71	0.26
DMR_25	0.31	0.16	0.77	0.41

Table S6. Motif enrichment analysis of the 67 hypomethylated regions in *SF3B1*^{mut} patients.

Rank	Motif Name	Motif	P-value	q-value (Benjamini)	# of Target Sequences with Motif (of 67)	% of Target Sequences with Motif	# of Background Sequences with Motif (of 42456)	% of Background Sequences with Motif
1	ZNF165 (Zf) WHIM12-ZNF165-ChIP-Seq (GSE65937)	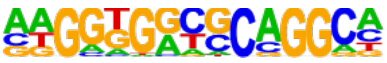	1.00E-03	0.398	9	13.43%	1600.6	3.77%
2	BHLHA15 (bHLH) NIH3T3-BHLHB8.HA-ChIP-Seq (GSE119782)	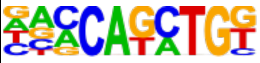	1.00E-02	0.5857	25	37.31%	9228.3	21.73%
3	Foxf1 (Forkhead) Lung-Foxf1-ChIP-Seq (GSE77951)	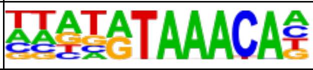	1.00E-02	0.5857	8	11.94%	1610.1	3.79%
4	Tcf12 (bHLH) GM12878-Tcf12-ChIP-Seq (GSE32465)	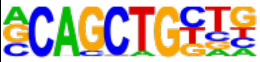	1.00E-02	0.5857	23	34.33%	8411.3	19.81%
5	Olig2 (bHLH) Neuron-Olig2-ChIP-Seq (GSE30882)	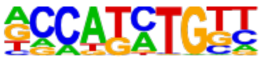	1.00E-02	0.5857	25	37.31%	9571.4	22.54%
6	Foxo3 (Forkhead) U2OS-Foxo3-ChIP-Seq (E-MTAB-2701)	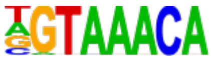	1.00E-02	0.5857	7	10.45%	1437.6	3.39%
7	Ascl1 (bHLH) NeuralTubes-Ascl1-ChIP-Seq (GSE55840)	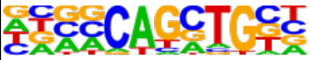	1.00E-02	0.5857	31	46.27%	13364.1	31.47%
8	AMYB (HTH) Testes-AMYB-ChIP-Seq (GSE44588)	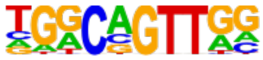	1.00E-02	0.5857	17	25.37%	5851.9	13.78%
9	FOXA1 (Forkhead) LNCAP-FOXA1-ChIP-Seq (GSE27824)	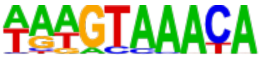	1.00E-02	0.5857	9	13.43%	2242.5	5.28%

Bibliography

1. Dietrich, S.; Oleś, M.; Lu, J.; Sellner, L.; Anders, S.; Velten, B.; Wu, B.; Hüllein, J.; da Silva Liberio, M.; Walther, T.; et al. Drug-perturbation-based stratification of blood cancer. *J. Clin. Invest.* **2018**, *128*, 427–445, doi:10.1172/JCI93801.
2. Lienhard, M.; Grasse, S.; Rolff, J.; Frese, S.; Schirmer, U.; Becker, M.; Börno, S.; Timmermann, B.; Chavez, L.; Sültmann, H.; et al. QSEA-modelling of genome-wide DNA methylation from sequencing enrichment experiments. *Nucleic Acids Res.* **2017**, doi:10.1093/nar/gkw1193.
3. Oakes, C.C.; Seifert, M.; Assenov, Y.; Gu, L.; Przekopowitz, M.; Ruppert, A.S.; Wang, Q.; Imbusch, C.D.; Serva, A.; Koser, S.D.; et al. DNA methylation dynamics during B cell maturation underlie a continuum of disease phenotypes in chronic lymphocytic leukemia. *Nat. Genet.* **2016**, doi:10.1038/ng.3488.
4. Binet, J.L.; Auquier, A.; Dighiero, G.; Chastang, C.; Piguet, H.; Goasguen, J.; Vaugier, G.; Potron, G.; Colona, P.; Oberling, F.; et al. A new prognostic classification of chronic lymphocytic leukemia derived from a multivariate survival analysis. *Cancer* **1981**, *48*, 198–206, doi:10.1002/1097-0142(19810701)48:1<198::aid-cncr2820480131>3.0.co;2-v.
5. Vollbrecht, C.; Mairinger, F.D.; Koitzsch, U.; Peifer, M.; Koenig, K.; Heukamp, L.C.; Crispatzu, G.; Wilden, L.; Kreuzer, K.A.; Hallek, M.; et al. Comprehensive analysis of disease-related genes in chronic lymphocytic leukemia by multiplex PCR-based next generation sequencing. *PLoS One* **2015**, doi:10.1371/journal.pone.0129544.
